# Heteroresistance Is Associated With *in vitro* Regrowth During Colistin Treatment in Carbapenem-Resistant *Klebsiella pneumoniae*

**DOI:** 10.3389/fmicb.2022.868991

**Published:** 2022-04-07

**Authors:** Yifan Wang, Xinqian Ma, Lili Zhao, Yukun He, Wenyi Yu, Shining Fu, Wentao Ni, Zhancheng Gao

**Affiliations:** Department of Pulmonary and Critical Care Medicine, Peking University People’s Hospital, Beijing, China

**Keywords:** *in vitro* regrowth, colistin, heteroresistance, *Klebsiella pneumoniae*, combination therapy

## Abstract

Polymyxins including polymyxin B and colistin (polymyxin E) are considered the last resort for treating infections caused by carbapenem-resistant gram-negative bacteria. However, *in vitro* regrowth with the emergence of resistance during treatment is common. Polymyxin heteroresistance, particularly in *Acinetobacter baumannii* and *Klebsiella pneumoniae*, has been widely reported. This study was primarily performed to evaluate the prevalence of colistin heteroresistance in carbapenem-resistant *K. pneumoniae* (CR-KP) and the association between *in vitro* regrowth and heteroresistance. The mechanisms of colistin resistance and the ability of combination therapies to suppress resistance selection were further investigated. A population analysis profile (PAP) analysis showed that 69 (71.9%) of 96 CR-KP strains had colistin heteroresistance. Time-kill assays revealed that the colistin monotherapy could quickly eliminate the bacterial cells in strains without heteroresistance within the first 6 h. Conversely, it could initially reduce the number of cells in heteroresistant strains, but then regrowth occurred rapidly. Resistance screening at 12 and 24 h in the time-kill assays indicated that susceptible populations were killed, and regrowth was the exact result of the continued growth of resistant subpopulations. Colistin resistance in the regrowth subpopulations was mainly due to the overexpression of *phoPQ* and *pmrD*. Colistin combined with tetracyclines (tigecycline or minocycline) or aminoglycosides (amikacin or gentamicin) could effectively suppress the resistance selection and significantly elicit *in vitro* synergistic effects. These findings suggested that the combination therapy can be used to treat infections caused by CR-KP with colistin heteroresistance. Nevertheless, further *in vivo* studies considering drugs pharmacokinetics/pharmacodynamics are needed to confirm these findings.

## Introduction

Polymyxins are now considered the last resort for the treatment of carbapenem-resistant gram-negative bacteria infections. Polymyxins, such as polymyxin B and colistin (polymyxin E), are cyclic cationic lipopeptides. They can bind to the lipopolysaccharides (LPS) of gram-negative bacteria and disrupt bacterial membranes, thereby exerting rapid bactericidal effects (Zhang et al., 2019). However, even with exposure to high bactericidal concentrations, regrowth with resistance development, and consequent treatment failure is quite common *in vitro* (Owen et al., 2007; Poudyal et al., 2008; Crémieux et al., 2019; Bian et al., 2021; Maynard et al., 2021). To investigate this phenomenon, a recent study used the hollow-fiber infection models and found that the total and bioactive polymyxin B concentrations in the culture media quickly declined within hours of treatment initiation and prior to bacterial regrowth (Maynard et al., 2021). They further observed that the regrowth in low-dose regimens was due to the amplification of strains that expressed low-level adaptive resistance. In contrast, regrowth at high dosages was due to the amplification of strains that expressed highly stable resistance.

The rapid decrease in drug concentrations below the target in culture broth may explain the occurrence of subsequent regrowth, but this phenomenon cannot fully explain the emergence of high resistance in the regrowth phase. Usually, the emergence of highly stable resistance is mainly associated with the pre-existent resistant subpopulations being picked under antibiotic treatment, and the evolution of resistance during treatment in some instances (Drlica and Zhao, 2007). The phenomenon of a larger antibiotic-susceptible bacterial population with small less-susceptible bacterial subpopulations is called heteroresistance (El-Halfawy and Valvano, 2015). Unlike stable genetic resistance, the clinical impact of heteroresistance is often underestimated or ignored because it cannot be detected by conventional antimicrobial susceptibility testing methods, such as minimum inhibitory concentration (MIC) determination (El-Halfawy and Valvano, 2015). For example, colistin heteroresistance has been widely reported in gram-negative bacteria, especially *Acinetobacter baumannii* and *Klebsiella pneumoniae* (Chen et al., 2020; Karakonstantis and Saridakis, 2020; Band et al., 2021; Seo et al., 2021; Tian et al., 2021). Two studies on *K. pneumoniae* strains with colistin heteroresistance have demonstrated the regrowth in all strains after several hours of exposure to colistin (Seo et al., 2021; Tian et al., 2021). However, further studies should investigate whether heteroresistance can fully explain the *in vitro* regrowth for polymyxins, and whether regrowth can also occur in purely susceptible strains without heteroresistance as an indication of the adaptive evolution of resistance.

Therefore, in this study, we compared the regrowth pattern between colistin-susceptible and heteroresistant carbapenem-resistant *K. pneumoniae* (CR-KP) to elucidate the role of heteroresistance in *in vitro* regrowth. Considering that two drugs with different resistance mechanisms may theoretically produce synergistic effects on bacterial killing and inhibition of resistance, we further evaluated the ability of colistin with tetracyclines or aminoglycosides to suppress *in vitro* regrowth.

## Materials and Methods

### Bacterial Strains and Minimum Inhibitory Concentration Determination

In our previous study, we tested the susceptibility of 168 non-duplicate CR-KP clinical strains collected from different patients during June 2014–December 2018 in four tertiary hospitals in Beijing, China (Ni et al., 2021). Among them, 98 CR-KP strains from three tertiary hospitals were used in this study. Most strains (53/98, 54.1%) were collected from the lower respiratory tract specimens, followed by the blood samples (31/98, 31.6%). If multiple strains were isolated from the same patient, only the first strain was chosen. Of the 98 strains, 88 (89.8%) produced the KPC-2 type carbapenemase, and 10 produced the NDM-1 type carbapenemase. A total of eight *K. pneumoniae* ST types were classified. ST11 was the most prevalent ST (69/98, 70.4%), followed by ST37 (13/98, 12.3%), ST395 (8/98, 8.2%), ST15 (3/98, 3.1%), ST48 (2/98, 2.0%), ST17 (1/98, 1.0%), ST23 (1/98, 1.0%), and ST290 (1/98, 1.0%). All strains were identified by the VITEK 2 Compact System (bioMérieux, Marcy-l’Étoile, France). *Escherichia coli* ATCC 25922 was used as a quality control strain in the MIC determination. The MICs for colistin and other antibiotics were determined by standard broth microdilution methods according to the recommendations of Clinical and Laboratory Standards Institute [CLSI], 2020. The breakpoint for colistin was based on the criteria established by the CLSI: The MIC ≤ 2 mg/L is judged as susceptible, and >2 mg/L as resistant.

### Population Analysis Profiling Assay

For screening the heteroresistance in CR-KP, population analysis profiling (PAP) assays were performed as previously described (Band et al., 2019). Strains were tested in three times to ensure the accuracy of the entire experiment. In brief, 0.5 McFarland overnight culture bacterial suspensions were serially diluted to bacterial suspensions from 10^8^ to 10^6^ CFU/ml, and 100 μl aliquots with different cell densities were plated onto Mueller–Hinton agar (MHA) plates with increasing concentrations of colistin (from the MIC to 128 mg/L). After incubation at 37°C for 48 h, strains were classified as heteroresistant if the number of colonies growing at 2.0- or 4.0-fold that of the breakpoint concentration was at least 0.0001% (1/10^6^) of those growing on antibiotic-free plates.

In addition, a modified PAP assay was used for examining the efficacy of colistin combined with tetracyclines (tigecycline or minocycline) or aminoglycosides (amikacin or gentamicin) on the suppression of heteroresistance. Bacterial suspensions (100 μl aliquots with cell densities of 10^8^ CFU/ml) were plated onto MHA plates with increasing concentrations of colistin (from the 1 × MIC to 128 mg/L) and a fixed concentration (1 × MIC) of the other drug. Following 48 h incubation at 37°C, the number of colonies was counted.

### Time-Kill Assay

The time-kill assays for colistin monotherapy and combination therapy were performed as previously described (Ni et al., 2021). The concentration of colistin and other antibiotics used in time-kill assays was 1 × MIC of each tested strain that was obtained from the susceptibility testing. Tubes containing 10 ml of freshly prepared cation-adjusted Mueller–Hinton broth (CAMHB) (Difco, Detroit, MI, United States) supplemented colistin alone or combined with other antibiotics were inoculated with Strains to a final density of approximately 5 × 10^6^ CFU/ml, and incubated with shaking at 37°C. Then, 100 μl aliquots of the culture sample were removed at 0, 3, 6, 9, 12, and 24 h, serially diluted and plated on antibiotic-free MHA plates for determining the total number of cells, or plated on agar plates containing different concentrations of colistin for determining the number of resistant subpopulations.

### Growth Curves

We selected one clinical strain K65 and its four resistant clones (named C1–C4) obtained from the PAP. After overnight culture, a 1:100 dilution of saturated culture was mixed into fresh Mueller–Hinton broth and shaken at 37°C for 3 h. After that, cultures were diluted in CAMHB to reach turbidity equal to 0.5 McFarland. Next, a 1:100 dilution of each 0.5 McFarland culture was mixed into CAMHB and shaken at 37°C continuously. The absorbance of the bacterial medium at 600 nm was measured every hour. Each strain was cultured in triplicate and the means absorbance was calculated.

### Quantitative Reverse Transcription PCR Analysis

Total bacterial RNA was extracted using the RNAprep pure cell/bacteria kit (Tiangen, Beijing, China) according to manufacturer instructions. cDNA was synthesized using the TIANScript cDNA kit (Tiangen, Beijing, China), and the expression of *phoP* and *pmrD* was quantified using a 7500 real-time PCR system (Applied Biosystems, Foster City, CA, United States). Housekeeping gene *rrsE* was used as an internal reference. Relative quantification of target genes (*phoP* and *pmrD*) was performed with the 2^–ΔΔCt^ method, normalized to *rrsE* and expressed as a relative fold change to *rrsE* expression.

### Whole Genome Sequencing and Transcriptome Analyses

We performed the WGS and transcriptome analyses for the parent strain K65 and its four heteroresistant clones mentioned above. For next-generation sequencing, the sequencing library was constructed by adding index codes to attribute sequences for each sample, with an average insert size of 474 bp. Paired-end reads of 150 bp were generated using the Illumina HiSeq platform at Shanghai Majorbio Bio-Pharm Technology Co., Ltd. Raw reads were filtered to remove low-quality reads (<Q20). Clean data then were assembled *de novo* using SOAPdenovo2 (Luo et al., 2012)^1^. Coding sequences of the genome were predicted using GeneMarkS, Glimmer^2^, and Prodigal software. The phylogenetic tree of K65 and four heteroresistant clones was constructed by comparsion with 31 house-keeping genes (*dnaG, frr, infC, nusA, pgk, pyrG, rplA, rplB, rplC, rplD, rplE, rplF, rplK, rplL, rplM, rplN, rplP, rplS, rplT, rpmA, rpoB, rpsB, rpsC, rpsE, rpsI, rpsJ, rpsK, rpsM, rpsS, smpB, and tsf*). Multilocus Sequencing Typing (MLST) analysis with the 7 housekeeping genes *gapA, infB, mdh, pgi, phoE, rpoB*, and *tonB* was performed to determine sequence types (STs) using the PubMLST database. The total RNA of each sample was extracted using the RNAprep Pure Bacteria Kit (Tiangen, China) following the manufacturer’s protocol. Ribosomal RNA (rRNA) was removed using the Ribo-Zero kit (Epicentre). RNA-Seq was undertaken using the Illumina HiSeq platform mentioned above. Several databases were used to predict gene functions, such as NR, Swiss-Prot, Pfam, EggNOG, Gene Ontology, and Kyoto Encyclopedia of Genes and Genomes. The RSEM software^3^ was used for the quantitative analysis of gene expression levels. DEGseq software was used to analyze the differentially expressed genes between groups by screening the threshold value of | log2FC| ≥ 1 and *p*-adjust < 0.05.

## Results

### Prevalence of Colistin Heteroresistance in Carbapenem-Resistant *K. pneumoniae*

**FIGURE 1:**
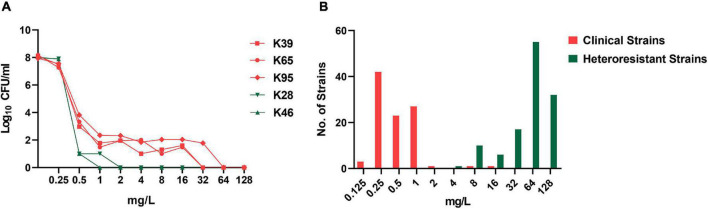
Population analysis profiling (PAP) for the identification of colistin heteroresistance and minimum inhibitory concentration (MIC) distribution of resistant subpopulations. **(A)** PAP of five representative strains with (K39, K65, and K95) or without (K28 and K46) colistin heteroresistance. **(B)** MIC distribution of clinical strains and resistant subpopulations selected in the PAP assays.

**FIGURE 2:**
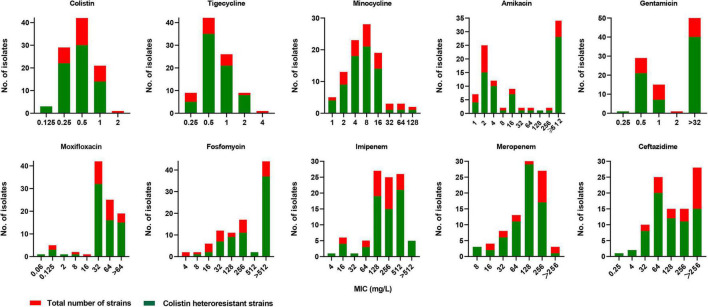
Distribution of colistin heteroresistance at different MICs of colistin, tigecycline, minocycline, amikacin, gentamicin, moxifloxacin, fosfomycin, imipenem, meropenem, and ceftazidime.

Among the 98 CR-KP strains, 2 (2/98, 2.0%) and 69 (69/96, 71.9%) showed colistin resistance (MIC > 2 mg/L) and colistin heteroresistance, respectively. The PAP results for five representative strains are shown in Figure 1A. For testing the stability of highest degrees of resistance in heteroresistant subpopulations, two heteroresistant colonies of each strain growing on MHA plates containing the highest drug concentrations were randomly selected from the consecutive five-generation cultures on antibiotic-free plates. These heteroresistant subpopulations showed stable high-MIC resistance to colistin. Their MICs were at least 8-fold higher than those of the parent strains, with MIC_50_ and MIC_90_ both of ≥16 mg/L (Figure 1B). The distribution of colistin heteroresistance at different MIC values of colistin and nine other antimicrobial agents is shown in Figure 2. The proportion of heteroresistant strains is uniformly distributed, suggesting no distinct associations between colistin heteroresistance and antibiotic resistance.

### Relationship Between Heteroresistance and *in vitro* Regrowth

**FIGURE 3:**
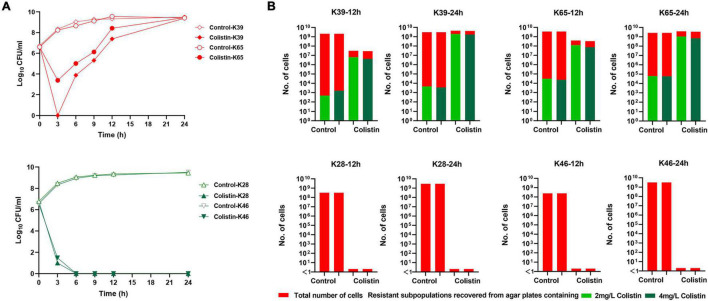
Time-kill curves of colistin monotherapy with 1 × MIC against carbapenem-resistant *Klebsiella pneumoniae* (CR-KP) strains and proportion of resistant subpopulations during the time-kill assays. (**A**,i) Time-kill curves of colistin monotherapy against two representative strains with colistin heteroresistance (K39 and K65). (**A**,ii) Time-kill curves of colistin monotherapy against two representative strains without colistin heteroresistance (K28 and K46). **(B)** Number of resistant subpopulations at 12 and 24 h in the control group and colistin monotherapy groups during time-killing assays. Bacteria were plated on the agar plates containing 2 and 4 mg/L colistin, and the resistant subpopulation of CFUs was counted.

We then examined the time-kill kinetics of colistin at 1 × MIC against all the strains. For heteroresistant strains, the number of cells decreased initially, but regrowth occurred quickly; for non-heteroresistant strains, the number of cells quickly decreased in the first 6 h without regrowth. The time-kill curves of the four representative strains are shown in Figure 3A. We further monitored resistance in the regrowth population. Four strains (two with colistin heteroresistance and two without heteroresistance) were randomly selected. Then, 100 μl aliquots of the culture sample were extracted at 12 and 24 h in the time-kill assays, serially diluted, and plated on MHA plates containing 2 or 4 mg/L colistin to determine the proportion of resistant subpopulations. As shown in Figure 3B, compared with the heteroresistant strains (K39 and K65), no resistant subpopulations of K28 and K46 recovered at 12 and 24 h (cell density 10^9^–10^10^ CFU/ml) in the control group (without colistin exposure). This result further confirmed that these strains were “purely susceptible” without heteroresistance. In the heteroresistant strains (K39 and K65), exposure to colistin significantly increased the frequency of resistance at 12 and 24 h.

### Homology Analysis and Growth Curves of Resistant Subpopulations

The whole genetic profile of four randomly selected resistant clones for K65 (C1–C4) were screened and compared with the parent strain. The parent strain and clones belonged to ST395, and the phylogenetic tree of 31 house-keeping genes showed high homology levels (Supplementary Figure 1A). To determine the difference in fitness costs between the susceptible strains and resistant subpopulations, we determined the growth curves of the parent strain (K65) and its four resistant clones (C1–C4). As shown in Supplementary Figure 1B, the growth rate had no significant differences between the resistant clones and parent strain, indicating that the resistant phenotype did not affect bacterial fitness.

### Mechanisms of Resistance in Colistin-Resistant Subpopulations

Some specific genes were found in the resistant clones but not in K65. Further analysis showed these clones shared several specific genes involved in gene transfer and recombination: *traV* encoding lipoprotein in the type IV conjugative transfer system, *rdgC* encoding recombination-associated protein RdgC, *insB* encoding IS1 family transposase and *umuC* encoding Y-family DNA polymerase (Supplementary Table 1). The two-component regulatory system *crrAB* was not identified, and no mutations in *phoP*, *phoQ*, *pmrB*, *pmrD*, *pmrA*, and *mgrB* were identified in the resistant clones.

**FIGURE 4:**
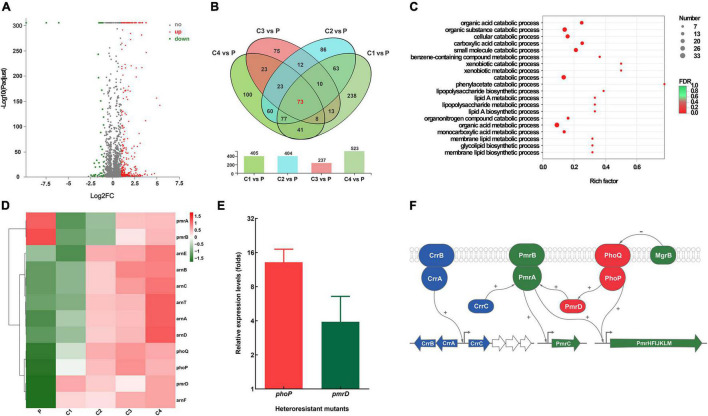
Comparative transcriptomic analysis between K65 (P) and four resistant clones (C1–C4). **(A)** Volcano plot showing upregulated (designated red square) and downregulated (designated green square) genes of the resistant clones. **(B)** Venn diagram of the differentially expressed genes obtained from the four resistant clones. **(C)** Gene Ontology (GO) enrichment analysis of differentially expressed genes. **(D)** Heat map clustering of differential expression of key genes associated with colistin resistance. **(E)** Relative expression of *phoP* and *pmrD* in colistin-resistant subpopulations. **(F)** Model of colistin resistance mechanisms.

We then compared the transcriptomic profiles of K65 and four resistant clones (C1–C4). Figure 4A showed the upregulated and downregulated genes in the resistant clones; Figure 4B showed the number of differentially expressed genes in the resistant clones. GO analysis revealed that the differentially expressed genes were mainly involved in biological processes, such as LPS biosynthesis and metabolism (Figure 4C). Further analysis revealed that resistant clones shared a similar pattern characterized by increased *phoPQ* and *pmrD* expressions levels relative to the parent strain (Figure 4D). The qRT-PCR of more strains and their resistant clones (*n* = 20) verified the findings (Figure 4E). Figure 4F shows the model of resistance mechanisms in the colistin-resistant subpopulations.

### Colistin Combination Therapy Effectively Suppressed *in vitro* Regrowth

**FIGURE 5:**
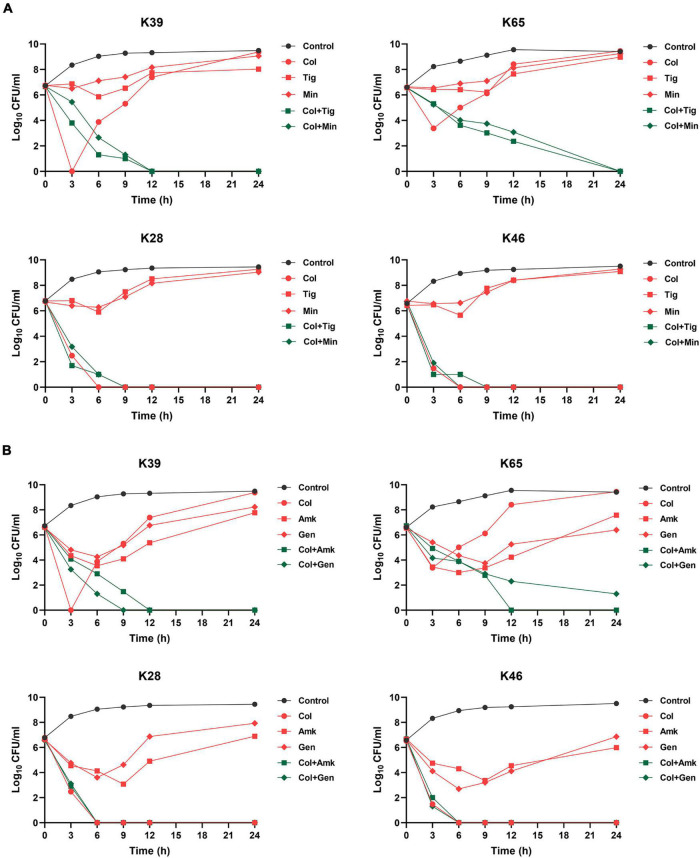
Time-kill curves of different combination therapies against representative CR-KP strains with (K39 and K65) or without (K28 and K46) colistin heteroresistance. **(A)** Time-kill curves of colistin combined with tigecycline or minocycline against representative CR-KP strains; Col, colistin; Tig, tigecycline; Min, minocycline. **(B)** Time–kill curves of colistin combined with amikacin or gentamicin against representative CR-KP strains; Amk, amikacin; Gen, gentamicin.

We further examined the efficacy of colistin combined with tetracyclines (tigecycline or minocycline) or aminoglycosides (amikacin or gentamicin) in suppressing heteroresistance. The modified PAP analysis showed that after another drug with 1 × MIC was added, no colonies recovered from agar plates containing 2 mg/L colistin in most strains (93/96). The time-kill curves verified the results of modified PAP for combination therapy. Four strains were randomly selected for the assays. As shown in Figure 5, for the heteroresistant strains (K39 and K65), combination therapies could constantly inhibit the growth of CR-KP, and no regrowth occurred within 24 h of treatment. For the non-heteroresistant strains (K28 and K46), colistin monotherapy could completely kill all the cells within 6 h, and the addition of the other drugs did not enhance colistin activity.

## Discussion

Polymyxins are recognized as a last resort therapeutic option for CR-KP infections. One important concern for the clinical use of polymyxins is heteroresistance, a phenomenon in which one genetically homogenous bacterial strain contains subpopulations with increased antibiotic resistance levels compared with that of the main susceptible population (El-Halfawy and Valvano, 2015). Colistin heteroresistance has been widely reported and its prevalence varies according to isolation sources, test methods and standards, and previous colistin exposure (Hawley et al., 2008). In our study, the prevalence of colistin heteroresistance in 98 CR-KP strains reached 71.9%. These resistant subpopulations showed the stable resistance to colistin, with MICs usually at least eight times higher than the breakpoints (2 mg/L).

We found that the colistin monotherapy could successfully eliminate the whole population, showing rapid killing effects in susceptible stains without heteroresistance. However, in heteroresistant strains, colistin monotherapy could initially reduce the number of bacteria in the culture media, but regrowth quickly occurred. Meanwhile, we observed that the frequency of resistant subpopulations significantly increased at 12 and 24 h, indicating that regrowth was an exact result of the continued growth of resistant subpopulations during colistin monotherapy. Therefore, the results of PAP and time-kill assays demonstrated that the heteroresistance is associated with the *in vitro* regrowth during polymyxin treatment, and the high prevalence of heteroresistance in CR-KP explains why regrowth is frequently reported.

Using genomic and transcriptomic analysis, we further analyzed the molecular mechanisms of resistance in colistin-resistant subpopulations by comparing the parental strain and resistant subpopulations. Colistin resistance is mediated by the addition of 4-deoxyaminoarabinose and/or phosphoethanolamine to lipid A, which causes an absolute increase in the lipid A charge, thereby reducing the affinity of positively charged colistin to LPS (Gogry et al., 2021). This process involves multiple molecular mechanisms, such as alternation in PmrAB, PhoPQ, and CrrAB two-component regulatory systems (Wright et al., 2015; Cheong et al., 2020; Yan et al., 2021), and inactivation or down-regulation of *mgrB* gene (Cannatelli et al., 2014; Poirel et al., 2015) and plasmid-mediated *mcr* (Hussein et al., 2021). Our study found that the significant mRNA overexpressions of *phoP*, *phoQ*, and *pmrD* in the resistant subpopulations were quite common, suggesting that the colistin resistance in CR-KP heteroresistant strains was mainly due to the upregulation of the PhoPQ two-component regulatory system.

Colistin heteroresistance can lead to the antibiotic treatment failure and cause the rapid emergence of resistance when using monotherapy against heteroresistant strains (Band et al., 2018). Combination therapy is a promising strategy for addressing the gap between the availability of new antibiotic drugs and the rising problem of colistin resistance. For example, colistin combined with rifampicin or tigecycline can effectively prevent the emergence of heteroresistance/resistance to colistin even at low concentrations (Gazel and Otkun, 2017). Polymyxin B combined with ceftazidime/avibactam can hamper the emergence of resistant subpopulations (Ma et al., 2019). Polymyxins combined with fosfomycin or tigecycline can overcome the heteroresistance in CR-KP (Crémieux et al., 2019; Tian et al., 2021). In our study, we evaluated the ability of colistin combined with tetracyclines or aminoglycosides to suppress the heteroresistance. In the PAP assays, with the addition of other drugs, the highest concentrations of colistin inhibiting resistant subpopulation growth could reduce to concentrations lower than the CLSI breakpoints (2 mg/L) in most heteroresistant strains. Time-kill assays further verified the findings of the PAP assays, showed that the synergistic effects of colistin combination therapies. However, for non-heteroresistant strains, the colistin combination therapy did not elicit the synergistic effects compared with monotherapy. Previous clinical studies obtained conflicting conclusions regarding colistin combination therapies, which are superior to monotherapy against the carbapenem-resistant gram-negative bacteria (Zusman et al., 2017; Durante-Mangoni et al., 2019). The different prevalence of colistin heteroresistance in these studies may affect the clinical efficacy of combination therapies. Heteroresistance screening with a rapid test before administrating colistin-based combination therapy for CR-KP infections may be needed. Generally, *in vitro* results suggest that colistin combination therapy may be a treatment strategy against heteroresistant CR-KP strains, but not against non-heteroresistant strains.

Our study has some limitations. Constant antibiotic concentrations were used in the *in vitro* study without considering pharmacokinetics. In addition, the *in vitro* study could not completely simulate *in vivo* environments, such as the exact ion concentrations and host immune responses. Therefore, further *in vivo* studies involving pharmacokinetic and pharmacodynamic investigations should be performed to optimize the dosage of colistin and combination therapies against heteroresistant CR-KP strains.

## Conclusion

Our study found that the regrowth occurred in colistin-heteroresistant strains but not in susceptible strains, indicating that selection of resistant subpopulations during polymyxin monotherapy is associated with *in vitro* regrowth. Colistin combined with tetracyclines or aminoglycosides can effectively suppress the selection of resistant subpopulations and show significant *in vitro* synergistic effects. Therefore, combination therapy may be used to treat infections caused by CR-KP with colistin heteroresistance.

## Data Availability Statement

The datasets presented in this study can be found in online repositories. The names of the repository/repositories and accession number(s) can be found below: https://www.ncbi.nlm.nih.gov/, PRJNA770945 and https://www.ncbi.nlm.nih.gov/, PRJNA770995.

## Ethics Statement

This study was an *in vitro* study and does not involve humans. The written informed consent was not required for this study according to the institutional ethical, biosecurity, and investigation committees because the bacterial strains in this study were obtained from hospital clinical laboratories.

## Author Contributions

YW, XM, and SF: data curation, formal analysis, investigation, methodology, project administration, and writing – original draft. LZ, YH, and WY: data curation, formal analysis, and methodology. WN: funding acquisition, data curation, formal analysis, investigation, supervision, and writing – review and editing. ZG: supervision and writing – review and editing. All authors contributed to the article and approved the submitted version.

## Conflict of Interest

The authors declare that the research was conducted in the absence of any commercial or financial relationships that could be construed as a potential conflict of interest.

## Publisher’s Note

All claims expressed in this article are solely those of the authors and do not necessarily represent those of their affiliated organizations, or those of the publisher, the editors and the reviewers. Any product that may be evaluated in this article, or claim that may be made by its manufacturer, is not guaranteed or endorsed by the publisher.

## Footnotes

1
https://github.com/aquaskyline/SOAPdenovo2

2
http://ccb.jhu.edu/software/glimmer/index.shtml

3
http://deweylab.github.io/RSEM/

## Supplementary Material

Supplementary Figure 1 Phylogenetic tree and growth curves of K65 and four resistant clones. **(A)** Phylogenetic tree of K65 and four resistant clones (C1–C4) constructed by comparsion with 31 house-keeping genes (*dnaG, frr, infC, nusA, pgk, pyrG, rplA, rplB, rplC, rplD, rplE, rplF, rplK, rplL, rplM, rplN, rplP, rplS, rplT, rpmA, rpoB, rpsB, rpsC, rpsE, rpsI, rpsJ, rpsK, rpsM, rpsS, smpB*, and *tsf*). **(B)** Growth curves of Kp65 and 4 resistant clones for 24 h.
