# Supplementary material for: Heteroresistance Is Associated With in vitro Regrowth During Colistin Treatment in Carbapenem-Resistant Klebsiella pneumoniae
Source: Front Microbiol. 2022 Apr 7;13:868991. doi: 10.3389/fmicb.2022.868991 (PMC9022032; doi:10.3389/fmicb.2022.868991)
Supplement: Supplementary file 1 [file Table_1.DOCX]

**Supplementary Table 1.** Specific genes (n=27) found in four heteroresistant clones but not in the parent isolate K65.

| Gene Name | Gene Description | C1 | C2 | C3 | C4 |
| --- | --- | --- | --- | --- | --- |
| *traV* | type IV conjugative transfer system lipoprotein TraV | **+** | **+** | **+** | **+** |
| *rdgC* | recombination-associated protein RdgC | **+** | **+** | **+** | **+** |
| *insB* | IS1 family transposase | **+** | **+** | **+** | **+** |
| *umuC* | Y-family DNA polymerase | **+** | **+** | **+** | **+** |
| *lamB* | maltoporin | **+** | **+** | **+** |  |
| *tolA* | hypothetical protein |  | **+** | **+** | **+** |
| *IS26* | IS6-like element IS26 family transposase |  | **+** | **+** |  |
| *frmR* | formaldehyde-responsive transcriptional repressor FrmR |  | **+** | **+** |  |
| *dmsB* | dimethylsulfoxide reductase subunit B | **+** |  | **+** |  |
| *ulaA* | PTS ascorbate transporter subunit IIC | **+** |  |  |  |
| *uhpB* | integral membrane sensor signal transduction histidine kinase | **+** |  |  |  |
| *ugpE* | ABC transporter permease | **+** |  |  |  |
| *tetR* | tetracycline resistance transcriptional repressor TetR(A) | **+** |  |  |  |
| *RPS18* | 40S ribosomal protein S18 | **+** |  |  |  |
| *plsB* | glycerol-3-phosphate 1-O-acyltransferase PlsB | **+** |  |  |  |
| *nhaA* | Na+/H+ antiporter NhaA | **+** |  |  |  |
| *lldP* | L-lactate permease | **+** |  |  |  |
| *impC* | type VI secretion system contractile sheath large subunit | **+** |  |  |  |
| *gsiA* | dipeptide transport ATP-binding protein DppD | **+** |  |  |  |
| *glgA* | glycogen synthase GlgA | **+** |  |  |  |
| *ghrB* | glyoxylate/hydroxypyruvate reductase GhrB | **+** |  |  |  |
| *galU* | GalU regulator GalF | **+** |  |  |  |
| *hofM* | pilus assembly protein |  | **+** |  |  |
| *hhoB* | outer membrane-stress sensor serine endopeptidase DegS |  | **+** |  |  |
| *mcp* | chemotaxis protein |  |  | **+** |  |
| *ssuC* | aliphatic sulfonate ABC transporter permease SsuC |  |  |  | **+** |
| *artJ* | transporter substrate-binding domain-containing protein |  |  |  | **+** |

**+** represents the gene was identified in the heteroresistant clone but not in the parent isolate.
