# Supplementary figures and images for: Heteroresistance Is Associated With in vitro Regrowth During Colistin Treatment in Carbapenem-Resistant Klebsiella pneumoniae
Source: Front Microbiol. 2022 Apr 7;13:868991. doi: 10.3389/fmicb.2022.868991 (PMC9022032; doi:10.3389/fmicb.2022.868991)

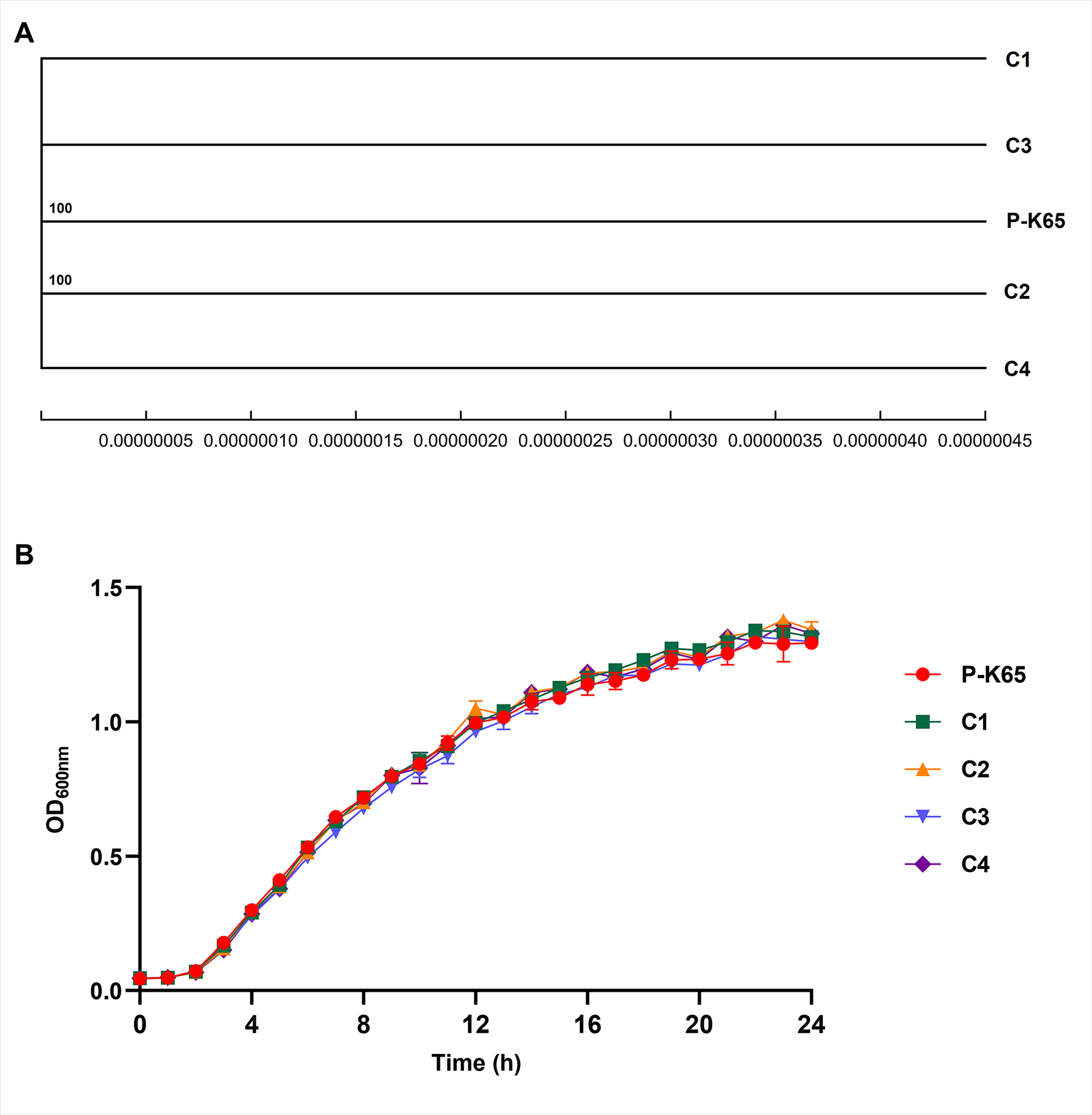

Supplement: Supplementary Figure 1 — Phylogenetic tree and growth curves of K65 and four resistant clones. (A) Phylogenetic tree of K65 and four resistant clones (C1–C4) constructed by comparsion with 31 house-keeping genes (dnaG, frr, infC, nusA, pgk, pyrG, rplA, rplB, rplC, rplD, rplE, rplF, rplK, rplL, rplM, rplN, rplP, rplS, rplT, rpmA, rpoB, rpsB, rpsC, rpsE, rpsI, rpsJ, rpsK, rpsM, rpsS, smpB, and tsf). (B) Growth curves of Kp65 and 4 resistant clones for 24 h. [file Image_1.TIF]
